# Prognostic value of transthoracic echocardiography score for the prognosis of continuous ambulatory peritoneal dialysis patients

**DOI:** 10.1186/s12882-024-03493-2

**Published:** 2024-02-23

**Authors:** Sheng Wan, Yanglin Hu, Li Cheng, Da He, Zengsi Wang, Yanmin Zhang

**Affiliations:** Department of Nephropathy, Wuhan No.1 Hospital, No. 215 of Zhongshan Avenue, 430030 Wuhan, China

**Keywords:** Transthoracic echocardiography score, Continuous ambulatory peritoneal dialysis, Mortality, Nomogram, End-stage kidney disease

## Abstract

**Background:**

We devoted ourselves to proving that the initial transthoracic echocardiography score (TTES) had predictive significance for patients with continuous ambulatory peritoneal dialysis (CAPD).

**Methods:**

In this retrospective analysis, 274 CAPD patients who had PD therapy were recruited sequentially. TTE exams were performed three months following the start of PD therapy. All patients were divided into two groups based on the strength of their TTES levels. TTES’s predictive value for CAPD patients was then determined using LASSO regression and Cox regression.

**Results:**

During a median of 52 months, 46 patients (16.8%) died from all causes, and 32 patients (11.7%) died from cardiovascular disease (CV). The TTES was computed as follows: 0.109 × aortic root diameter (ARD, mm) − 0.976 × LVEF (> 55%, yes or no) + 0.010 × left ventricular max index, (LVMI, g/m^2^) + 0.035 × E/e’ ratio. The higher TTES value (≥ 3.7) had a higher risk of all-cause death (hazard ratio, HR, 3.70, 95% confidence index, 95%CI, 1.45–9.46, *P* = 0.006) as well as CV mortality (HR, 2.74, 95%CI 1.15–19.17, *P* = 0.042). Moreover, the TTES had an attractive predictive efficiency for all-cause mortality (AUC = 0.762, 95%CI 0.645–0.849) and CV mortality (AUC = 0.746, 95%CI 0.640–0.852). The introduced nomogram, which was based on TTES and clinical variables, exhibited a high predictive value for all-cause and CV mortality in CAPD patients.

**Conclusion:**

TTES is a pretty good predictor of clinical outcomes, and the introduced TTES-based nomogram yields an accurate prediction value for CAPD patients.

**Supplementary Information:**

The online version contains supplementary material available at 10.1186/s12882-024-03493-2.

## Introduction

Continuous ambulatory peritoneal dialysis (CAPD) has become one of the most widespread renal replacement therapies (RRT) among individuals with end-stage kidney disease (ESKD), particularly for patients in remote areas, due to the significant benefits of residual kidney function protection, treatment-related accessibility, and reduced healthcare costs [1–3]. However, the average mortality rate of PD patients remains unacceptably high in recent decades, and the predominant cause of death is cardiovascular disease (CVD), which kills almost half of all PD patients [4–6]. As a result, accurate sensors or an algorithm for prediction for detecting initially PD patients who are at an increased risk of mortality may lead to successful amelioration in monitoring modalities and so improve PD patients’ prognosis.

Ultrasound has been extensively employed to either directly or indirectly access the interior and exterior makeup of patients’ hearts, kidneys, and other organs. Prior works have discovered the diagnostic and prognostic significance of ultrasound characteristics for a variety of disorders [7–9]. Transthoracic echocardiography (TTE), which was formerly used mostly on the heart, has received increased attention in recent years for renal illness. A recent study found that various 2-dimensional speckle-tracking echocardiography measures showed diagnostic relevance for acute kidney damage (AKI) after cardiac surgery [10]. Despite the acute renal disease, TTE values have been linked to the prognosis of patients with chronic kidney disease (CKD), ESKD, and kidney transplantation [11, 12]. However, little research has been attempted to explore the interaction between integrated TTE characteristics and the long-term survival of CAPD patients. As a result, in this study, we created a novel TTE score (TTES) based on real-world data in CAPD patients at our hospital and evaluated its predictive significance. Furthermore, for the prognosis of CAPD patients, dynamic sequence diagrams based on the TTES were created.

## Materials and methods

### Patients and study design

**Fig. 1:**
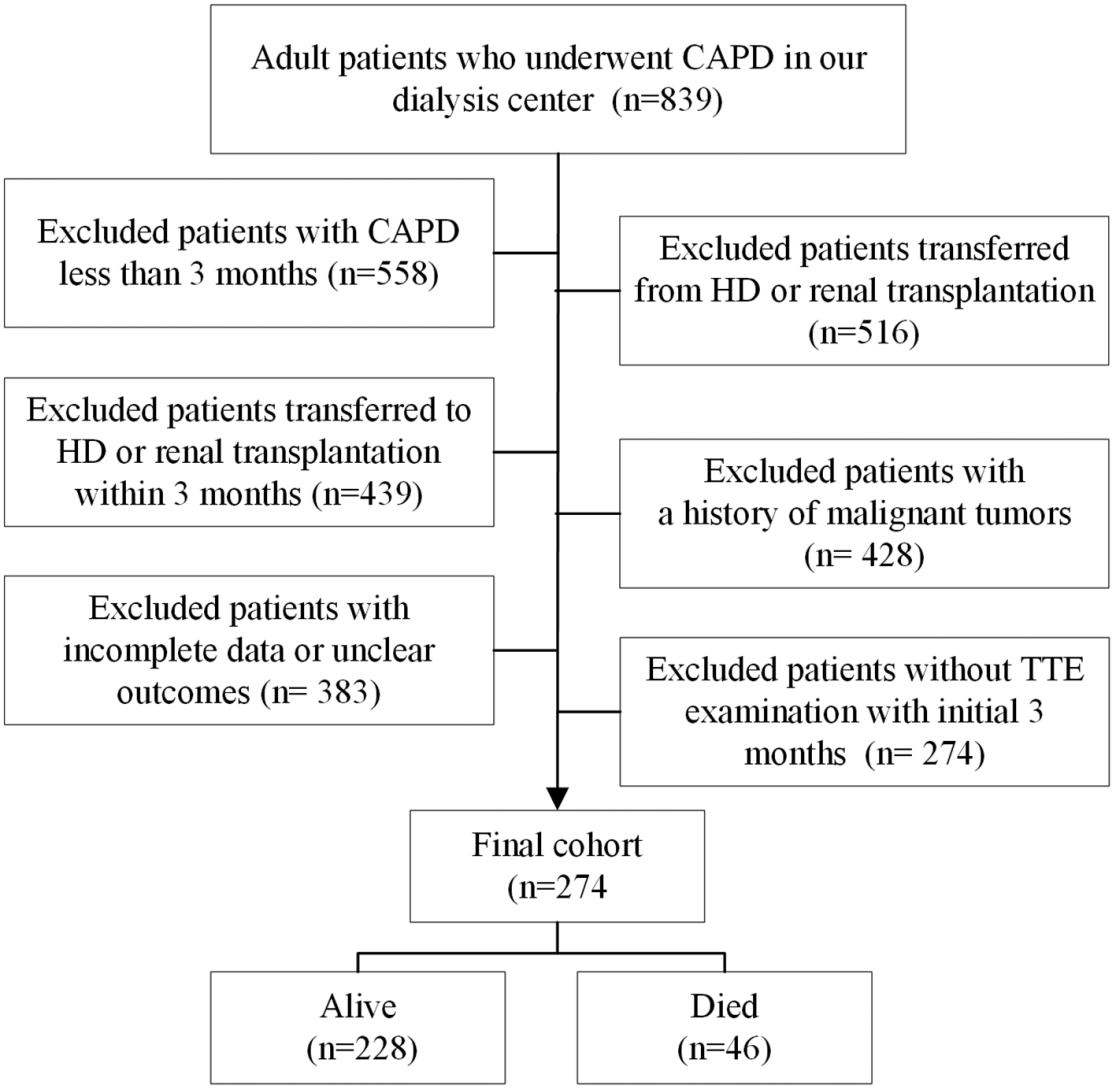
The flow chart of this study

From 1 March 2010 to 31 December 2022, participants in this single-site retrospective cohort study employed constant CAPD as their first RRT and underwent a catheter inserted in the PD center at Wuhan No.1 hospital. Our dialysis clinic is one of the biggest in the middle of China, with over 400 PD patients and more than 700 hemodialysis (HD) patients getting treatment at our facility regularly. This study included the following categories of patients: [1] adult patients who had just begun PD treatment, [2] patients who had been receiving PD treatment in our facility for more than three months, and [3] patients who had no fewer than one TTE assessment within the first three months of starting PD therapy. We further eliminated patients who were moved from HD or kidney transplantation, transferred to other PD clinics, got PD treatment within 3 months of receiving HD or kidney transplantation, and had previous diagnoses of malignancies. Furthermore, we excluded individuals who did not have a TTE measurement or who had incomplete critical baseline information (missing rate > 20%). Finally, this investigation included 274 PD patients (Fig. 1).

This study was approved by the Ethics Committee of Wuhan No. 1 Hospital without the need for informed consent from the participants.

### Patients’ characteristics

Baseline variables such as socio-demographics, marital and educational status, major causes of ESKD, complications, drug consumption, laboratory findings, and PD-related indicators were gathered from our PD database and medical records. Laboratory data were obtained at our hospital’s biochemical laboratory using conventional procedures, and all analyses were taken within three months following the start of PD treatment. Cardiovascular disease was classified as ischemic heart disease, cardiac failure, or peripheral vascular disease.

### Echocardiographic parameters

Within three months of the start of PD treatment, TTE exams were carried out by skilled echocardiographers using commercial ultrasound equipment (EPIQ 5, Philips Medical Imaging, Andover, USA). According to the recommendations of the American Society of Echocardiography (ASE), all measures and pictures were taken [13–15]. According to the most recent ASE recommendations, the following measurements were made in the long-axis parasternal view: standard aortic root diameter (ARD), ascending aorta diameter (AAOD), left ventricular end-diastolic diameter (LVEDD), septum thickness diameter (IVSD), left atrial end-systolic diameter (LAESD), left ventricular posterior wall (LVPW), and left ventricular fractional shortening (LVFS). Based on ASE guidelines, the left ventricular maximum index (LVMI) was created to calculate the left ventricular ejection fraction (LVEF) and left ventricular mass (LVM) and index them to the body surface area. In accordance with the recommendations of ASE, the standard right atrial diameter (RAD), right ventricular diameter (RVD), and RV outflow tract diameter (RVOT) at the end of diastole were all measured in the apical 4-chamber view and left parasternal short-axis view, respectively. Pulsed-wave spectral Doppler tissue pictures from the same view on the septal side of the mitral annulus were used to assess the peak early (E) and late (A) transmitral inflow velocities and early (e’) and late (a’) diastolic mitral annular peak velocities. Normal ventricular systolic function was established as an LV ejection fraction of more than 55%.

### Outcomes

The all-cause mortality was the major end-point, and the CV mortality was the secondary endpoint. Every patient was checked on at least once a month using our center’s database of medical information or outpatient dialysis clinics. All patients were monitored through December 31, 2022, or until they died, were switched to hemodialysis, underwent a kidney transplant, or were censored.

### Statistical analysis

The statistical program R (version 4.1.0) was used for all computations. The COX analysis’s coefficients were used to produce the novel TTES, which was then grown into two groups relying on the X-tile software’s ideal cutoff value. By using the least absolute shrinkage and selection operator (LASSO) regression, every one of the possible mortality factors was evaluated. The predictive nomogram for CAPD patients was subsequently created using univariate and multivariate COX regression to provide a quick and comparatively accurate tool for clinicians. The area under the receiver operating characteristic curves (AUC), calibration plots, and decision curve analysis (DCA) were used to evaluate the performance of this nomogram. P 0.05 was regarded as a significant value.

## Results

### Patients’ characteristics

Last but the least, 274 patients were recruited in this retrospective investigation, with 146 (53.3%) men and 128 (46.7%) women, and a mean age of 51.8 13.2 years. Few patients (48, 17.5%) received a college education, and most patients (239, 87.2%) were married when they began their initial PD medication. In our CAPD patients, hypertension was the most prevalent comorbidity (238, 86.9%), followed by diabetes (77, 28.1%), and 47 (17.2%) of our participants had a history of CVD. Furthermore, chronic glomerulonephritis (134, 48.9%) was the most prominent leading cause of ESKD, after diabetes (60, 21.9%), hypertension (51, 18.6%), and other or unrecognized causes (29, 10.6%). In Table 1, all patients’ details are shown. Simply put, elderly patients died more frequently and had decreased hemoglobin, platelets, blood albumin, and more substantial hsCRP amounts. In terms of echocardiographic measures, dead patients exhibited higher ARD, LVMI, RAD, e’, and E/e’ ratios and worse LVEF and LVFS (Table 2).

**Table 1:** Baseline characteristics of all patients in this study

Characteristics	Survival (*n* = 228)	Death (*n* = 46)	P value
Age, years old	50.6 ± 13.1	58.0 ± 12.2	< 0.001
Gender, male, n (%)	118 (51.8)	28 (60.9)	0.333
Body mass index, kg/m^2^	23.1 ± 3.7	22.5 ± 2.9	0.321
**Marital status, n (%)**			0.250
Unmarried	32 (14.0)	3 (6.5)	
Married	196 (86.0)	43 (93.5)	
**Education status, n (%)**			0.480
Illiterate/primary	38 (16.7)	12 (26.1)	
Middle school	87 (38.2)	17 (37.0)	
Secondary school	62 (27.2)	10 (21.7)	
University or above	41 (18.0)	7 (15.2)	
**Cause of ESRD, n (%)**			0.215
Chronic glomerulonephritis	114 (50.0)	20 (43.5)	
Diabetic nephropathy	48 (21.1)	12 (26.1)	
Hypertensive nephropathy	39 (17.1)	12 (26.1)	
Others or unknown	27 (11.8)	2 (4.3)	
**Comorbidities, n (%)**			
Cardiovascular disease	23 (10.1)	24 (52.2)	< 0.001
Hypertension	198 (86.6)	40 (87.0)	1.000
Diabetes	62 (27.2)	15 (32.6)	0.572
**Treatments, n (%)**			
ACEI/ARB	140 (61.4)	23 (50.0)	0.203
βblockers	109 (47.8)	24 (52.2)	0.203
CCB	192 (84.2)	36 (78.3)	0.442
Diuretics	12 (5.3)	1 (2.2)	0.604
Statins/fibrates	22 (9.6)	5 (10.9)	1.000
**Dialysis dose**			
Weekly total Ccr	76.8 ± 18.3	56.7 ± 16.5	0.550
Weekly renal Ccr	22.6 ± 8.3	16.6 ± 6.4	0.140
Weekly total Kt/Vurea	1.8 ± 0.5	1.7 ± 0.5	0.194
Weekly renal Kt/Vurea	0.3 ± 0.1	0.3 ± 0.2	0.424
PET at baseline	0.74 ± 0.13	0.70 ± 0.15	0.105
**Laboratory results**			
White blood cells, × 10^9^/L	7.0 ± 2.0	7.3 ± 3.3	0.293
Hemoglobin, g/L	110.0 ± 19.9	101.5 ± 22.7	0.011
Platelets, × 10^9^/L	218.8 ± 63.6	196.2 ± 65.8	0.029
Albumin, g/L	35.4 ± 4.1	26.8 ± 3.4	0.010
Blood urea nitrogen, umol/L	19.3 ± 5.0	18.1 ± 5.1	0.134
Serum creatinine, umol/L	986.9 ± 197.5	937.5 ± 192.9	0.304
Total cholesterol, mmol/L	4.5 ± 1.0	4.4 ± 1.2	0.583
Triglyceride, mmol/L	1.9 ± 0.9	2.2 ± 1.0	0.186
HDL-C, mmol/L	1.2 ± 0.4	1.1 ± 0.3	0.120
LDL-C, mmol/L	2.2 ± 0.8	2.0 ± 0.7	0.100
Calcium, mmol/L	2.3 ± 0.2	2.4 ± 0.2	0.507
Phosphorus, mmol/L	1.7 ± 0.4	1.7 ± 0.5	0.922
iPTH, pg/mL	435.2 ± 144.5	565.5 ± 273.3	0.101
hsCRP, mg/L	6.4 ± 1.6	9.9 ± 3.7	0.043
Dialysis duration, months	29.5 (5.5, 70.0)	25.5 (2.0, 59.5)	0.659
Urine volume, mL	300.0 (0.0, 837.5)	200.0 (0.0, 750.0)	0.753
PET at baseline	0.8 ± 0.2	0.7 ± 0.2	0.364

ESRD, end-stage renal disease, ACEI/ARB, angiotensin converting enzyme inhibitors/angiotensin receptor blocker, CCB, calcium calcium blockers, HDL-C, high-density lipoprotein cholesterol, LDL-C, low-density lipoprotein cholesterol, PET, peritoneal equilibration test, iPTH, intact parathyroid hormone, CRP, c-reactive protein

**Table 2:** Echocardiographic characteristics of all patients in this study

Characteristics	Survival (*n* = 228)	Death (*n* = 46)	P value
ARD, mm	23.6 ± 3.9	27.7 ± 4.7	< 0.001
AAOD, mm	32.2 ± 4.1	31.7 ± 3.4	0.503
LVEDD, mm	48.1 ± 8.5	47.4 ± 7.6	0.611
LAESD, mm	37.1 ± 5.6	38.8 ± 6.9	0.072
IVSD, mm	10.6 ± 2.2	11.2 ± 1.9	0.100
LVPW, mm	10.5 ± 6.1	10.2 ± 1.8	0.794
LVEF, %	60.0 ± 8.3	54.0 ± 10.2	< 0.001
LVFS, %	32.7 ± 6.5	30.9 ± 5.6	0.090
LVM, g	189.8 ± 76.1	192.9 ± 76.0	0.823
LVMI, g/m^2^	107.0 ± 34.8	123.4 ± 46.9	0.007
RAD, mm	33.7 ± 5.9	36.2 ± 6.0	0.010
RVD, mm	21.1 ± 3.9	21.7 ± 3.1	0.304
RVOT, mm	25.1 ± 4.1	25.0 ± 4.0	0.863
**Pulsed wave doppler**			
E, cm/s	71.4 ± 26.1	68.8 ± 18.2	0.519
A, cm/s	91.0 ± 23.8	95.8 ± 17.2	0.195
E/A ratio	0.93 ± 0.34	0.74 ± 0.22	0.293
**Tissue doppler**			
e’, cm/s	7.0 ± 2.8	6.1 ± 2.2	0.046
E/e’ ratio	10.9 ± 3.0	14.1 ± 4.5	< 0.001

ARD, aortic root diameter, AAOD, ascending aorta diameter, LVEDD, left ventricular end-diastolic dimension, LAESD, left atrial end-systolic diameter, IVSD, interventricular septal thickness, LVPW, left ventricular posterior wall thickness, LVEF, left ventricular ejection fraction, LVFS, left ventricular fraction shortening, LVM, left ventricular mass, LVMI, left ventricular mass index, RAD, right atrial diameter, RVD, right ventricular diameter, RVOT, right ventricular outflow tract

### Transthoracic doppler echocardiography score (TTES)

**Fig. 2:**
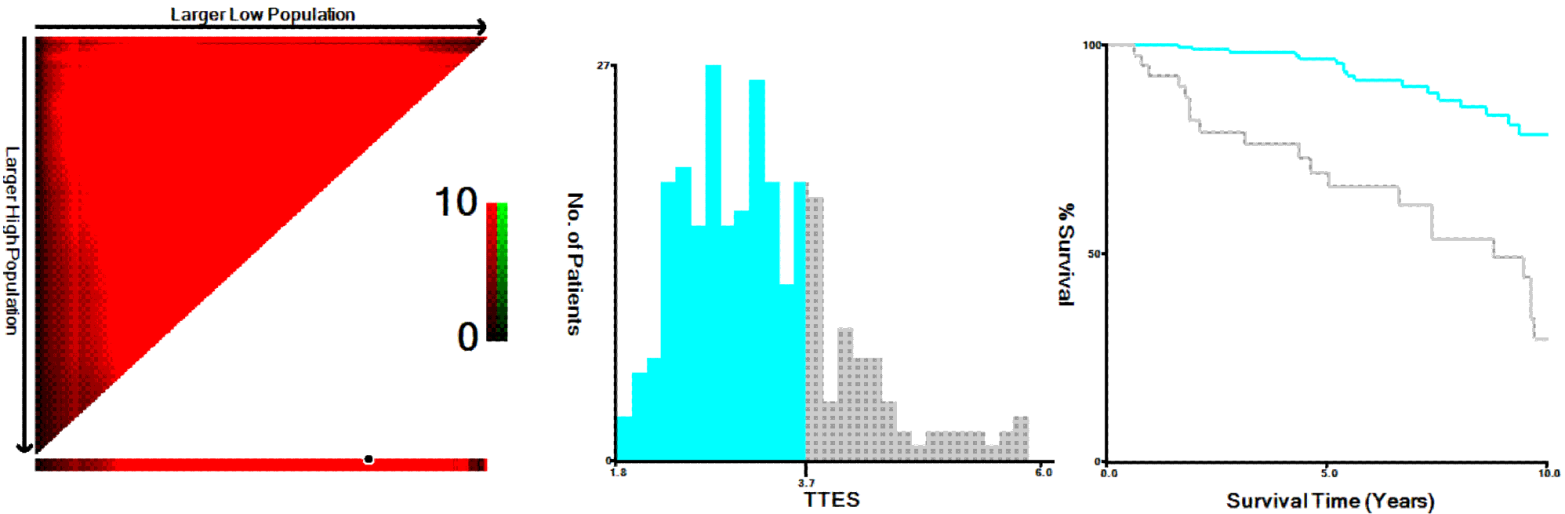
X-tile analyses of TTES to obtain the optimal cutoff value of TTES. X-tile plots for patients with CAPD are shown on the left panels; the black circles indicate the optimal cutoff values, which are also presented in histograms (middle panels). Kaplan-Meier curves are shown in the right panels

**Fig. 3:**
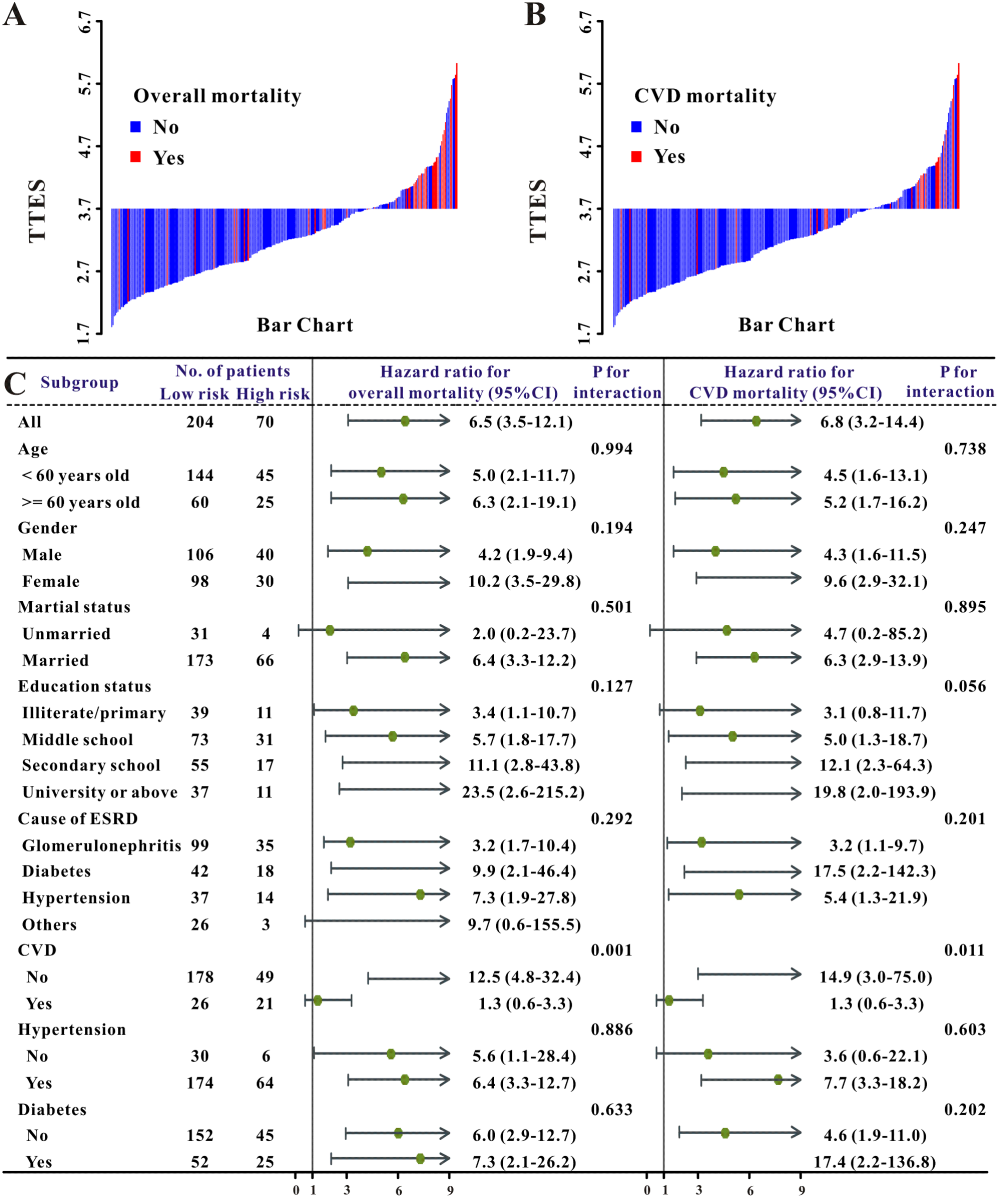
The waterfall plots and forest plots of the high-TTES group and low-TTES group for the prognosis of CAPD patients. The waterfall plot of TTES for each patient of all-cause mortality (**A**), CVD mortality (**B**), and the subgroup analysis of the TTES for the prognosis of individuals with CAPD patients (**C**)

The elements obtained from TTE were used to compute the innovative TTES using univariable and multivariable COX regression analysis. Ultimately, the unique TTES was calculated using ARD, LVEF 55%, LVMI, and E/e’ ratio. As a result, the novel TTES was constructed using the coefficients as follows: ARD (mm) × 0.109 - LVEF (> 55%, yes or no) × 0.976 + 0.010 × LVMI (g/m^2^) + 0.035 E/e’ ratio (Table 3). All patients were put into two groups predicated on the TTES value (3.7) obtained from the X-tile program (Fig. 2): high TTES (> 3.7) and low TTES (≤ 3.7) groups. Moreover, the TTES for patients treated was shown as a waterfall plot, with significant differences between alive and dead patients (*P* < 0.001, Fig. 3A–C). TTES levels were substantially connected with parameters relevant to PD treatment as well as other clinical characteristics, as illustrated in Supplemental Fig. 1.

**Table 3:** COX regression models of transthoracic echocardiography parameters for overall mortality of CAPD patients

	Univariate			Multivariate		
	β	HR (95%CI)	P	β	HR (95%CI)	P
ARD	0.113	1.120 (1.067–1.175)	< 0.001	0.109	1.116 (1.058–1.188)	0.001
AAOD	0.047	1.048 (0.972–1.130)	0.219			
LVEDD	0.000	1.000 (0.967–1.034)	0.991			
LAESD	0.038	1.038 (0.999–1.079)	0.057			
IVSD	0.223	1.249 (1.090–1.432)	0.001	0.190	1.209 (0.990–1.478)	0.063
LVPW	0.005	1.005 (0.961–1.052)	0.822			
LVEF ≥ 55	-1.486	0.226 (0.111–0.463)	< 0.001	-0.976	0.377 (0.166–0.854)	0.019
LVFS	-0.053	0.948 (0.900-1.000)	0.049	-0.031	0.970 (0.915–1.028)	0.301
LVM	0.002	1.002 (0.999–1.005)	0.219			
LVMI	0.010	1.010 (1.004–1.016)	0.001	0.010	1.010 (1.004–1.016)	0.002
RAD	0.039	1.040 (0.995–1.087)	0.080			
RVD	0.032	1.032 (0.964–1.105)	0.367			
RVOT	0.006	1.006 (0.937–1.080)	0.866			
E	-0.004	0.996 (0.984–1.008)	0.519			
A	0.007	1.007 (0.995–1.020)	0.246			
E/A ratio	-1.417	0.242 (0.057–1.028)	0.054			
e’	-0.150	0.860 (0.751–0.985)	0.030	-0.016	0.984 (0.847–1.143)	0.831
E/e’ ratio	0.074	1.077 (1.032–1.124)	0.001	0.035	1.036 (1.007–1.098)	0.040

CAPD, continuous ambulatory peritoneal dialysis, HR, hazard ratio, 95%CI, 95% comfidence interval, ARD, aortic root diameter, AAOD, ascending aorta diameter, LVEDD, left ventricular end-diastolic dimension, LAESD, left atrial end-systolic diameter, IVSD, interventricular septal thickness, LVPW, left ventricular posterior wall thickness, LVEF, left ventricular ejection fraction, LVFS, left ventricular fraction shortening, LVM, left ventricular mass, LVMI, left ventricular mass index, RAD, right atrial diameter, RVD, right ventricular diameter, RVOT, right ventricular outflow tract

**Fig. 4:**
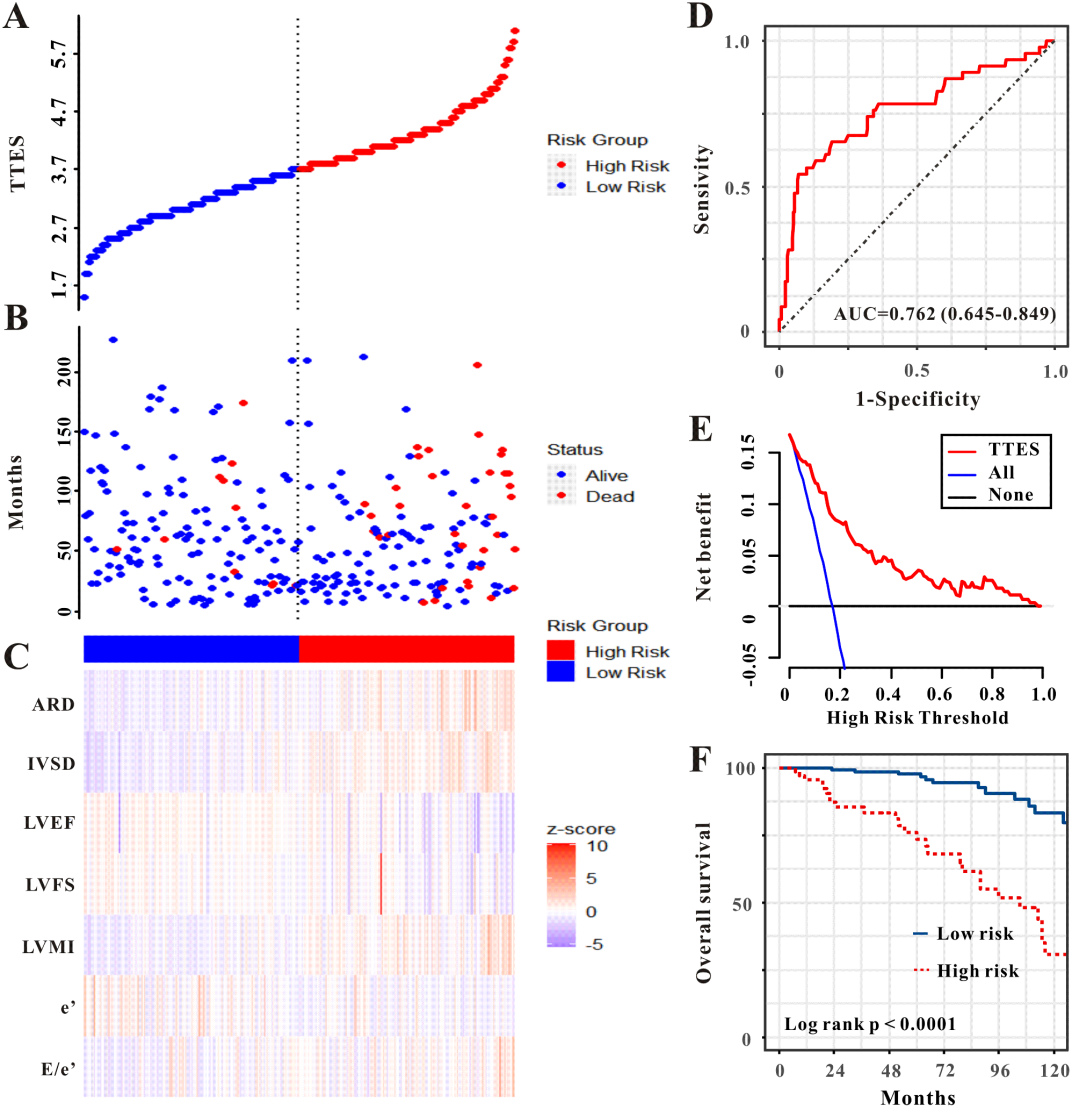
The TTES was established to detect the overall mortality of patients with CAPD. All patients were distinguished into high and low risk based on the TTES (**A**), the relationship between survival time and all-cause mortality of patients in the two corresponding groups (**B**), and the heatmap of other markers between the two groups (**C**). Receiver operating characteristic (ROC) curve analysis of the TTES for overall mortality (**D**), Decision curve analysis of the TTES for the overall mortality (**E**). Kaplan-Meier curves show the overall mortality of groups with different risks (**F**)

During a median follow-up duration of 52 months, 46 patients (16.8%) died from all causes and 32 patients (11.7%) died from CV disorders. In addition, even after correcting for other medical information, patients in the high TTES group had a greater risk of all-cause death (hazard ratio, HR, 3.70, 95% confidence index, 95%CI, 1.45–9.46, *P* = 0.006) as well as CV mortality (HR, 2.74, 95%CI 1.15–19.17, *P* = 0.042) (Table 4; Fig. 4A–C, and Supplemental Fig. 2A–C), and the crude HR was 2.03 (95%CI 1.54–2.67, *P* < 0.001), 2.08 (95%CI 1.50–2.89, *P* < 0.001), when the TTES value was utilized for continuous covariates.

**Table 4:** Univariate and multivariate COX regression analysis for clinical outcomes of high risk group and low risk group

Methods	HR (95%CI)	P value
**For overall mortality**		
Unadjusted	6.52 (3.51–12.11)	< 0.001
Adjusted for model I	3.89 (1.88–8.02)	< 0.001
Adjusted for model II	4.40 (1.90–10.20)	0.001
Adjusted for model III	3.70 (1.45–9.46)	0.006
**For cardiovascular mortality**		
Unadjusted	6.80 (3.21–14.37)	< 0.001
Adjusted for model I	3.41 (1.44–8.07)	0.005
Adjusted for model II	3.39 (1.16–9.92)	0.026
Adjusted for model III	6.70 (1.15–39.17)	0.035

HR, hazard ratio, 95%CI, 95% confidence index, Model I adjusted for age, gender, body mass index, martial status, education status. Model II adjusted for model I plus primary cause, comorbidities and treatment. Model III adjusted for model II plus dialysis dose and laboratory results

The TTES was also found to have an attractive predictive efficiency for all-cause mortality and CV mortality, with AUCs of 0.762 (95% CI 0.645–0.849, sensitivity, 64.4%, specificity, 83.0%) and 0.746 (95% CI 0.640–0.852, sensitivity, 63.1%, specificity, 81.3%), respectively (Fig. 4D, and Supplemental Fig. 2D). Nonetheless, DCA revealed that TTES was clinically beneficial for all-cause and CV mortality (Fig. 4E, and Supplemental Fig. 2E). The high-TTES group had a worse prognosis for patients with CAPD than the lower-TTES group (Fig. 4F, and Supplemental Fig. 2F, *P* < 0.0001).

### Development and verification of the predictive nomogram

**Fig. 5:**
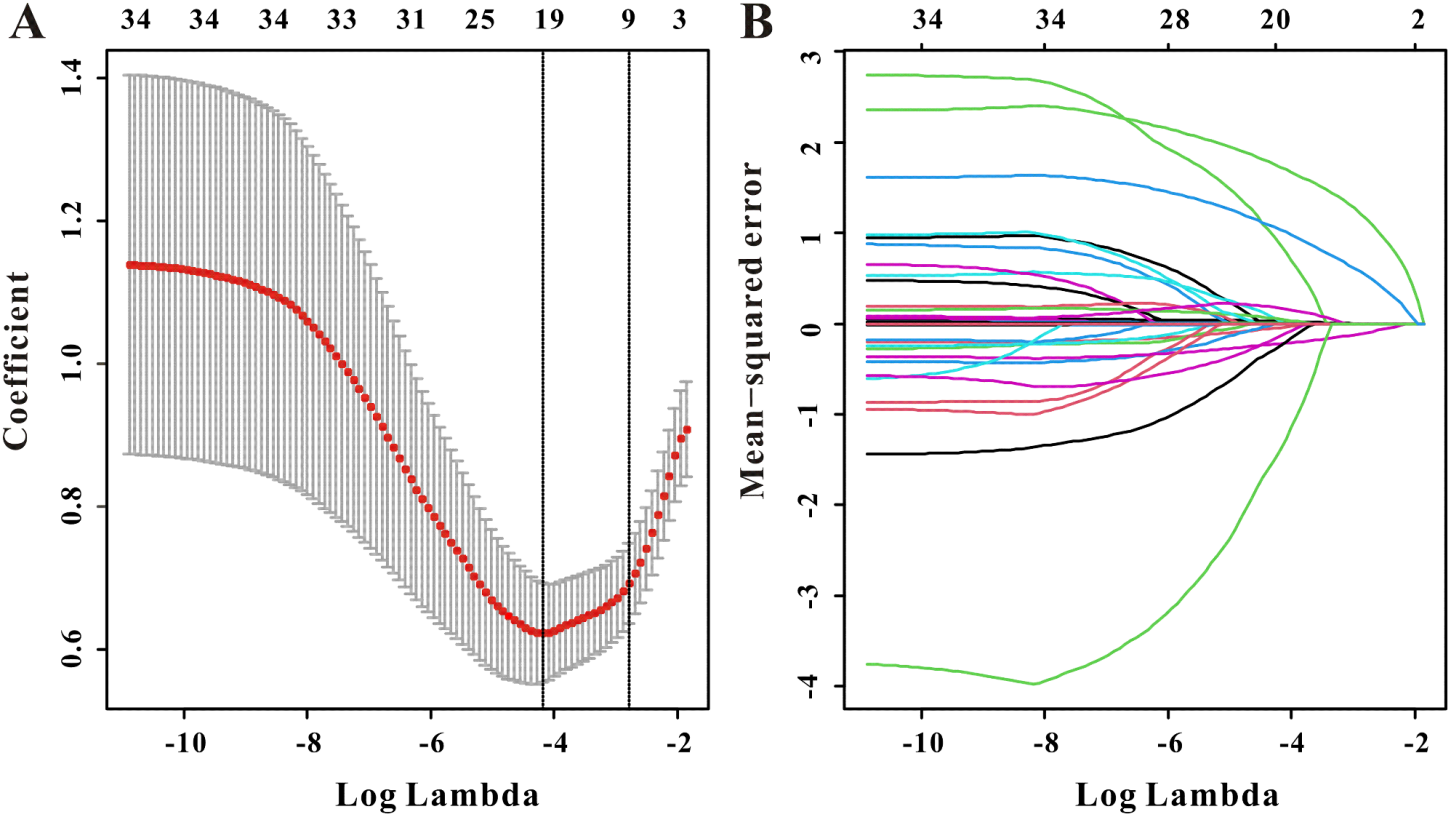
Selection of significant factors associated with all-cause mortality in CAPD patients by LASSO COX regression model

**Fig. 6:**
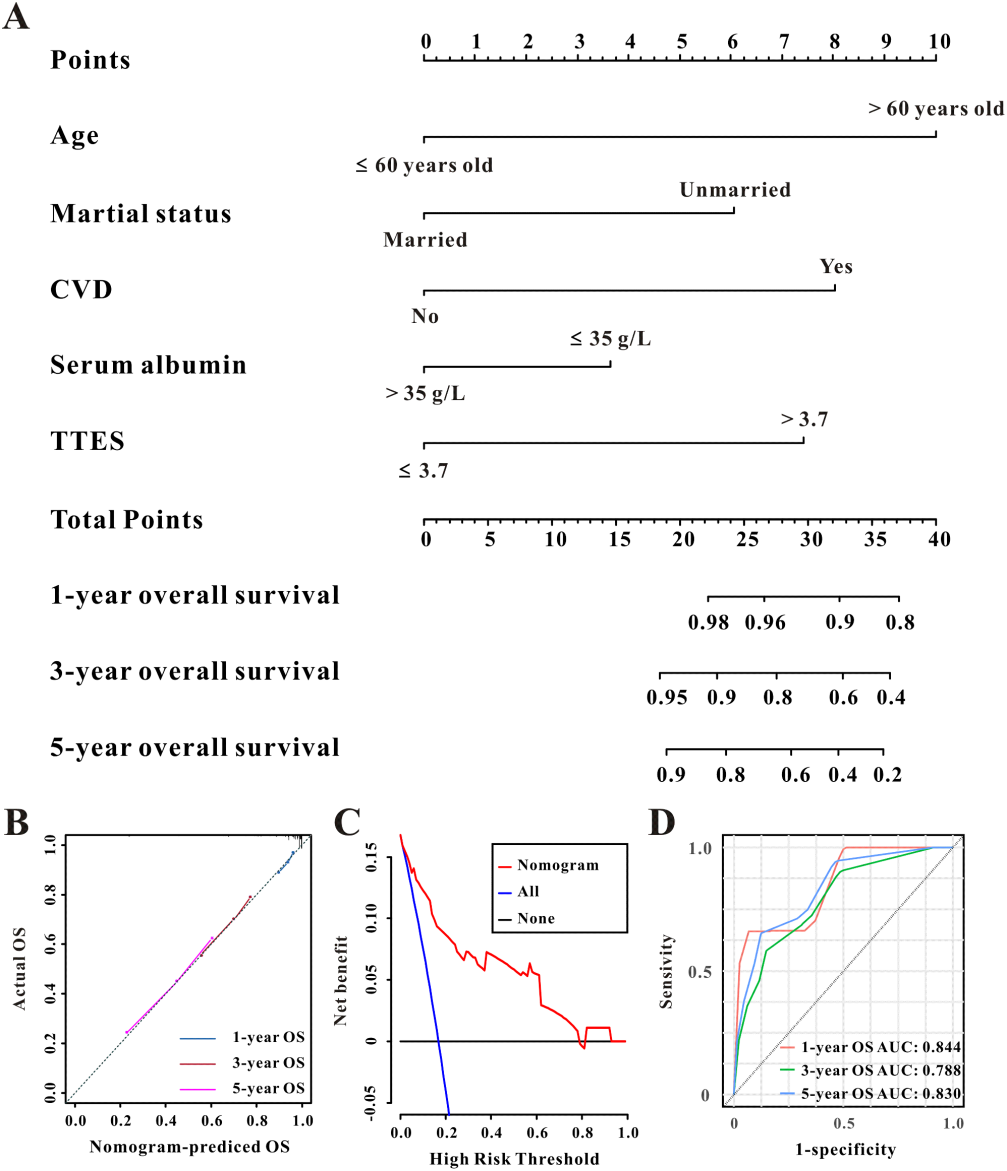
The established nomogram for all-cause mortality in patients with CAPD (**A**), the 1-year, 3-year, and 5-year calibration curves of the nomogram for the overall survival **(B)**, Decision curve analysis of the nomogram for overall survival (**C**), and the time-dependent ROC curves for the 1-year, 3-year, and 5-year overall survival in patients with CAPD (**D**)

The LASSO COX regression analysis chose 9 variables with nonzero coefficients for all-cause mortality, as shown in Fig. 5A-B. COX regression was also used to further sift predictors because of the small sample size and delivering a portable tool with comparatively high precision for doctors. Age, marital status, CVD, serum albumin, and TTES were eventually enlisted to create the prediction nomogram for all-cause mortality (Fig. 6A), as described in Table 5.

**Table 5:** COX regression analysis for the predictors of overall survival selected by LASSO regression

	Univariate analysis		Multivariate analysis	
	HR (95%CI)	P value	HR (95%CI)	P value
**Age**				
≤ 60 years old	Ref.	-	Ref.	-
> 60 years old	6.57 (3.53-12,23)	< 0.001	5.01 (2.50-10.03)	< 0.001
**Martial status**				
Married	Ref.	-	Ref.	-
Unmarried	3.97 (1.23–12.86)	0.021	4.23 (1.06–16.90)	0.041
**Cardiovascular disease**				
Yes	5.13 (2.87–9.18)	< 0.001	3.09 (1.51–6.33)	0.002
No	Ref.	-	Ref.	-
**Hemoglobin**				
≤ 90 g/L	Ref.	-	Ref.	-
> 90 g/L	0.49 (0.26–0.93)	0.029	0.72 (0.32–1.61)	0.417
**Serum albumin**				
≤ 35 g/L	Ref.	-	Ref.	-
> 35 g/L	0.35 (0.20–0.63)	< 0.001	0.86 (0.78–0.95)	0.004
Blood urea nitrogen	0.99 (0.94–1.05)	0.820		
Triglyceride	1.02 (0.86–1.22)	0.788		
**iPTH**				
≤ 300 umol/L	0.52 (0.29–0.95)	0.032	0.78 (0.39–1.55)	0.481
> 300 umol/L	Ref.	-	Ref.	-
**TTES**				
≤ 3.7	Ref.	-	Ref.	-
> 3.7	6.52 (3.51–12.11)	< 0.001	3.17 (1.63–6.14)	0.001

HR, hazard ratio, 95%CI, 95% confidence interval, iPTH, intact parathyroid hormone, TTES, transthoracic echocardiography score

Additionally, the forecasting nomogram’s 1-, 3-, and 5-year AUC for all-cause mortality was 0.844, 0.788, and 0.830, respectively (Fig. 6D). The calibration curves likewise showed a high level of agreement between projected and actual mortality (Fig. 6B). Furthermore, DCA indicated that the prediction nomogram was useful for decision-making in CAPD patients for all-cause mortality (Fig. 6C).

## Discussion

In this retrospective investigation, we initially formulated a unique TTES for the prognosis of first CAPD patients using real-world information from 274 patients at our dialysis clinic, and we found that the TTES had an adequate predictive ability for all-cause mortality and CV mortality. Furthermore, a straightforward and portable nomogram involving TTES and clinical traits was created and authorized for mortality with excellent calibration, discrimination, and clinical assistance. As a consequence, those findings revealed that initial TTES was a reliable prognostic predictor for CAPD patients, and the predictive nomogram based on TTES strength produced an accurate prediction for CAPD risk stratification.

Despite enormous gains in knowledge and facilities over the past decades, the prognosis of patients with CAPD remained consistently poor, and the main contributory cause of death was CVD, which varied from 48 to 70% in prior studies [16, 17]. Song et al. conducted a multi-center retrospective research of 586 CAPD patients and discovered that the overall mortality rate was 8.7%, with CVD accounting for about 60.0% (51/85) of all-cause deaths [18]. A recent study of 188 CAPD patients found that the overall death rate was 39.9% (75/188) after a median of 60 months of follow-up [19]. In comparison to the previous research, we included 274 CAPD patients in our dialysis facility and discovered that total mortality was 15.3%, with approximately 69.6% (32/46) of deceased patients dying from CVD, which was consistent with earlier studies. As a result, a reliable and innovative biomarker or prediction model for the mortality of CAPD patients may be developed for doctors to risk stratify, and then early and effective medicines might be implemented to improve these patients’ prognoses and lead to decreased healthcare expenditures.

Although ultrasound has been widely used to directly or indirectly access the architecture and functioning of patients’ hearts and kidneys, little research has looked at the varied parameters of ultrasound for the diagnosis and prognosis of various kidney illnesses. Hu et al. conducted an observational analysis of 23,945 eligible AKI patients using data from a public database and found that prompt TTE was related with improved clinical outcomes for intensive care unit patients [20]. Furthermore, Guo et al. discovered that TTE characteristics might be used to increase the accuracy of AKI diagnosis in patients who underwent cardiac valve surgery [21]. Ultrasound has been used to care for individuals with CKD in addition to acute renal disease. Jahn et al. conducted a study of 285 CKD patients and 34 age-matched healthy people (no kidney or CVD) who performed 2D speckle tracking echocardiography (STE) and found that STE could detect reduced LVEF in CKD patients, which could be used to predict all-cause mortality and CV mortality [22]. Another study, which enrolled 3505 CKD patients, found that baseline TTE parameters were independently associated with an increased risk of subsequent heart failure morbidity and mortality and that they could obtain marginal incremental prognostic facility over clinical factors for CKD patients [23].

TTE may be a cost-effective and even regular evaluation for patients with ESKD because of its simple and quickly accessible approach. CVD and kidney disease have multiple similar bidirectional pathways, which is also known as cardiorenal syndrome. As a result, physicians focused on the use of TTE and their parameters for CKD complications and even ESKD based on the notion that TTE or their values might play diagnostic and predictive roles in various illnesses. Sun et al. observed that LVMI and E/e’ ratio were resilient for all-cause mortality and CV mortality even after controlling for certain other variables in a 3-year prospective analysis of 181 patients on maintenance hemodialysis [24]. Our study contributed to the data that LVMI and E/e’ ratios obtained from TTE might be used to risk stratify individuals with ESKD. In patients with CAPD, Ye et al. confirmed that ARD and IVSd had predictive values for patients on stable PD medication [25]. Although prior research has revealed a correlation between TTE data and mortality in several renal conditions, the reasons behind this link may be multifactorial. Moreover, in this study, we found a significant correlation between TTES and other clinical factors, hemoglobin and serum albumin, for example, which might give an explanation that TTES could partly reflect the nutritional status of these CAPD patients. Aside from the aforementioned TTE parameters, we discovered a positive correlation between ARD and clinical outcomes, and we considered the possible reasons for this phenomenon as follows: the amplification of ARD is associated with refactoring of the aortic wall, which may lead to aortic artery stiffness and degeneration of aortic function, and thus these patients may result in poor clinical outcomes.

This retrospective research has some drawbacks. To begin, it was a retrospective, single-center study with small sample size, and we did not divide the patients into two separate groups, instead relying on bootstrap resampling to validate our nomogram. Second, we only enrolled the first TTE parameter readings; these values may alter throughout their follow-up at our facility, affecting the patient’s clinical results. Moreover, we did not include some other important factors for the prognosis of CAPD patients, the therapy for secondary hyperparathyroidism, and so on. Finally, because the study’s sample was limited to CAPD patients, whether the findings applicable to patients on hemodialysis or ESKD without dialysis should be validated in future investigations.

## Conclusions

In this work, we initially determined that the TTES, a simple and inexpensive measure, may serve as a reliable marker for CAPD patients, with good calibration, discrimination, and clinical assistance. Furthermore, the provided nomogram on the strength of TTES might serve as a viable instrument for identifying CAPD patients at high risk of death and could provide a therapeutic tip for CVD patients.

### Electronic supplementary material

Below is the link to the electronic supplementary material.

**Supplementary Material 1**: Supplementary figures

## Data Availability

The datasets used in this study are available from the corresponding author.
