# Supplementary material for: Prognostic value of transthoracic echocardiography score for the prognosis of continuous ambulatory peritoneal dialysis patients
Source: BMC Nephrol. 2024 Feb 23;25:65. doi: 10.1186/s12882-024-03493-2 (PMC10893662; doi:10.1186/s12882-024-03493-2)
Supplement: Supplementary file 1 — Supplementary Material 1: Supplementary figures [file 12882_2024_3493_MOESM1_ESM.pdf]

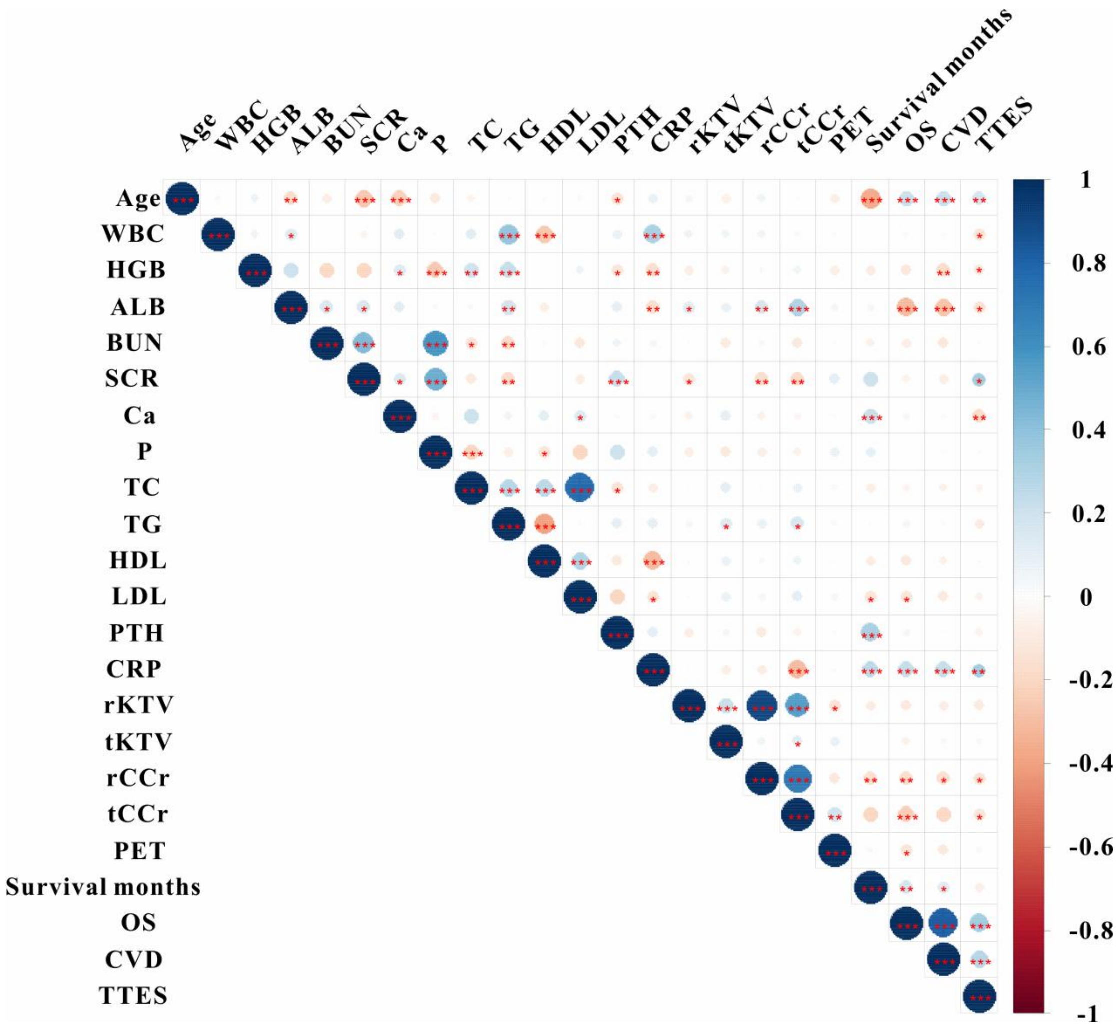

Supplemental Figure 1 The correlations between TTES and other clinical variables, and clinical outcomes. WBC, white blood cells, HGB, hemoglobin, ALB, albumin, BUN, blood urea nitrogen, SCR, serum creatinine, TC, Total cholesterol, TG, Triglyceride, HDL, high-density lipoprotein cholesterol, LDL, low-density lipoprotein cholesterol, PTH, intact parathyroid hormone, CRP, c-reactive protein, rKTV, renal KT/Vurea, tKTV, total KT/Vurea, OS, overall survival, CVD, cardiovascular death, TTES, transthoracic echocardiography score.

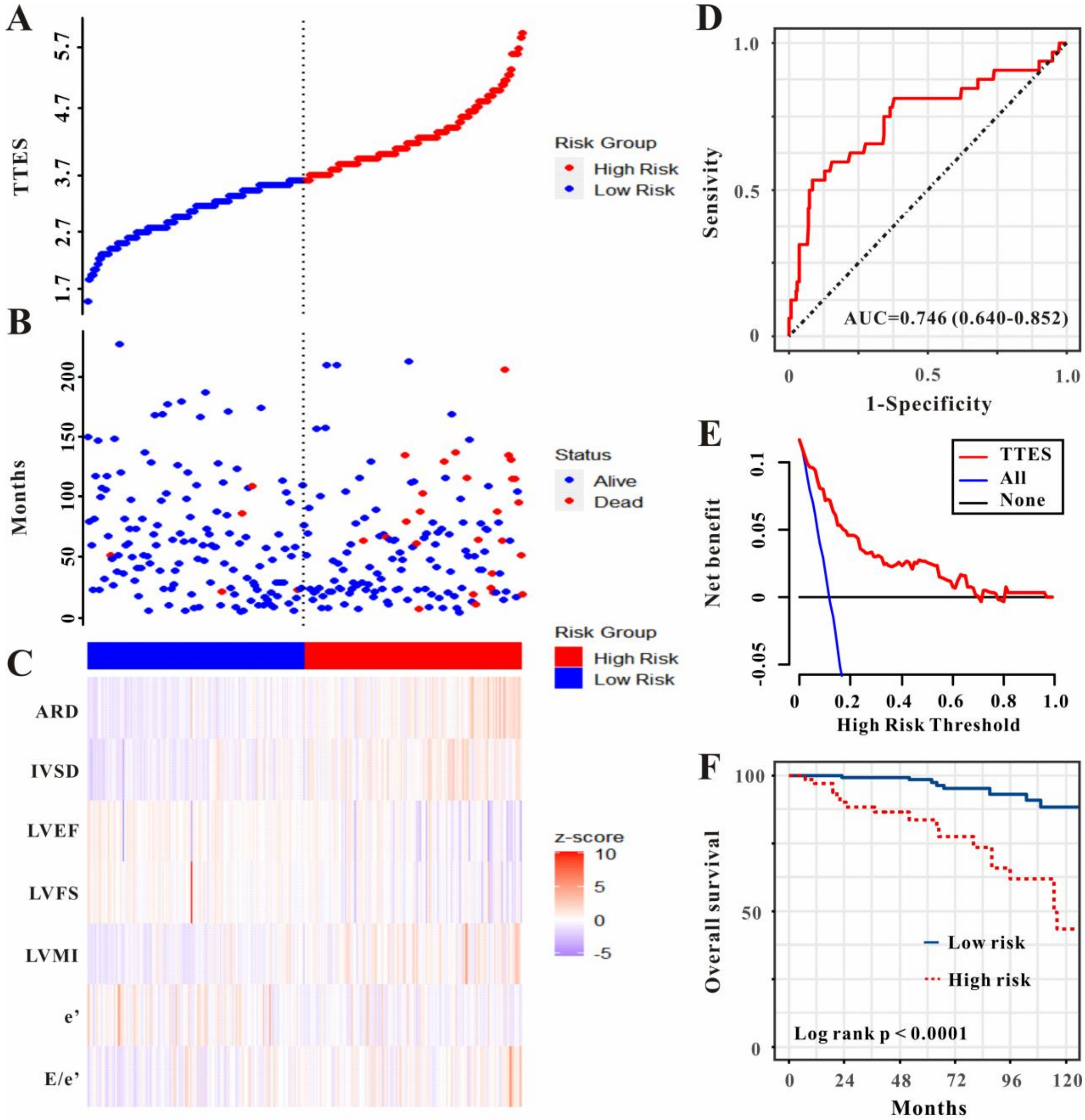

Supplemental Figure 2 The TTES was established to detect the CVD mortality of patients with CAPD. All patients were distinguished into high and low risk based on the TTES (**A**), the relationship between survival time and CVD mortality of patients in the two corresponding groups (**B**), and the heatmap of other markers between the two groups (**C**). Receiver operating characteristic (ROC) curve analysis of the TTES for CVD mortality (**D**), Decision curve analysis of the TTES for the CVD mortality (**E**). Kaplan-Meier curves show the CVD mortality of groups with different risks (**F**).
